# The relationship between perceived classroom climate and academic performance among English-major teacher education students in Guangxi, China: The mediating role of student engagement

**DOI:** 10.3389/fpsyg.2022.939661

**Published:** 2022-08-04

**Authors:** Yan Ma, Changwu Wei

**Affiliations:** ^1^School of Foreign Studies, Hezhou University, Guangxi, China; ^2^College of Education and Music, Hezhou University, Guangxi, China; ^3^International College, Dhurakij Pundit University, Bangkok, Thailand

**Keywords:** perceived classroom climate, student engagement, academic performance, English-major teacher education students, SD-R model

## Abstract

The academic performance of teacher education students predicts their future career development and it is also a significant factor related to their future students’ academic performance. However, little is known about the associations between perceived classroom climate, student engagement and academic performance, especially for English-major teacher education students. This study was to examine English-major teacher education students’ academic performance in relation to perceived classroom climate, student engagement. The questionnaire consisted of the Emotional Classroom Climate Scale, the Utrecht Work Engagement Scale – Student Form-3 Item (UWES-SF-3), and the 5-item Academic Performance Scale. This study investigated 307 English-major teacher education students in Guangxi, China. Among them, 280 (91.2%) were females, and 27 (8.8%) were males, aged between 18 and 24 (*M* = 20.34, SD = 1.26). Results indicated that perceived classroom climate was positively associated with student engagement and academic performance among English-major teacher education students; their student engagement was positively associated with their academic performance, and student engagement partially mediated the relationship between perceived classroom climate and academic performance. The findings supported the motivational process of study demands-resources (SD-R) model and revealed that perceived classroom climate and student engagement were significant factors linked to academic performance, and practical suggestions were discussed.

## Introduction

China began to carry out teacher education professional certification in 2017 (MOE of PRC, 2017b). In the context of teacher education reform, teacher education colleges have been trying to explore practical ways for the cultivation and training of teacher education students (Zhou and Chen, 2021). Studies have demonstrated that excellent teachers are crucial to students’ academic success (Hanushek, 2011; Goldring et al., 2014). Recently, domestic studies also revealed that the quality and skills of teachers are associated with students’ academic performance. For example, teachers’ emotional intelligence, job engagement and self-efficacy (Wang, 2022), perceived teacher support (Tao et al., 2022), and teacher feedback (Ma et al., 2022) are related to students’ academic performance. As future teachers, teacher education students need to learn relevant professional knowledge systematically and make corresponding professional learning and skill preparation according to the professional requirements (Zhang et al., 2011). In addition, as academic performance can predict students’ future career development (Negru-Subtirica and Pop, 2016; Van der Aar et al., 2019), teacher education students’ academic performance deserves attention.

From the existing domestic research, there is no operational definition of academic performance for Chinese teacher education students. The Ministry of Education of China has set clear accreditation standards for secondary education majors (MOE of PRC, 2017a) and teachers’ professional competence standards for secondary education majors (MOE of PRC, 2021). According to the purpose of this study, we defined teacher education students’ *academic performance* as students’ performance in educational and subject knowledge and professional competence concerning professional ethics values, teaching practice competence, comprehensive education competence, and competence in reflection and professional growth. There have been plenty of studies on the academic performance of college students both in China and abroad (May and Elder, 2018; Mao et al., 2022; March-Amengual et al., 2022; Tafesse, 2022; Wang, 2022). However, studies on teacher education students’ academic performance seem insufficient, especially those of English-major teacher education students. In the context of teacher education reform in China, it is necessary to pay attention to the academic performance of teacher education students. Therefore, this study took a sample of English-major teacher education students to explore the characteristics of their academic performance and its associations with perceived classroom climate and student engagement.

As Moos (1980) asserted, the classroom is an essential locus for student personal and academic growth, and classrooms have distinct climates that mediate student growth. Therefore, classroom climate may be one of the factors associated with students’ academic performance. Researchers have proposed diverse operational definitions of classroom climate. Nevertheless, these definitions all relate to teacher-student interactions (Wang et al., 2020). According to Hong et al. (2021), classroom emotional climate should include four dimensions regarding academic support from teachers, promoting interaction, promoting mutual respect, and respect for viewpoints. Based on this concept, we defined *perceived classroom climate* as students’ perception of the classroom climate concerning these four dimensions. Many studies have shown that classroom climate predicts academic performance (Johnson, 2006; Gutiérrez et al., 2019; Jafari and Asgari, 2020). Similarly, some domestic studies also revealed that classroom climate is an important factor related to academic performance. For example, a recent study of Chinese adolescents in Shandong province revealed that the teacher-student relationship is positively associated with students’ academic performance (Ma et al., 2022). Li et al. (2021a) argued that teacher-support and good teacher-student relationships are related to better self-control, which promotes academic performance. Nonetheless, another study by Mohamed et al. (2018) reported that classroom climate has no significant association with academic performance. This finding suggests that other factors may mediate the relationship between classroom climate and academic performance. Therefore, this study intended to explore student engagement as a mediator between perceived classroom climate and academic performance.

*Student engagement* is “a positive and satisfactory state of mind described as vigor, dedication, and absorption” (Schaufeli et al., 2002). It has been seen as an essential factor related to positive academic performance (Vahala and Winston, 1994; Mirzaei-Alavijeh et al., 2016; Ayala and Manzano, 2018; Jafari and Asgari, 2020; Tomaszewski et al., 2020). According to Dimitriadou et al. (2021), student engagement promotes academic performance and is positively associated with on-time graduation. Meanwhile, studies both in China and abroad have revealed that the perceived classroom climate is positively associated with student engagement (Rubie-Davies, 2015; Gutiérrez et al., 2019; Lu et al., 2022). In addition, it has been demonstrated that student engagement often plays a partial or complete mediating effect on the association between academic performance and other variables, such as social support (Chen and Chen, 2021; Siu et al., in press), perceived efficacy (Chong et al., 2018; Wang, 2022), teaching style, learning environment and socioeconomic status (Simpson and Burnett, 2019; Tomaszewski et al., 2020; Song et al., 2022). However, little is known about the mediating effect of student engagement on the association between perceived classroom climate and academic performance. Therefore, this study intended to examine student engagement as a mediator between these two variables.

## Research framework and hypotheses

### Research framework

**FIGURE 1:**
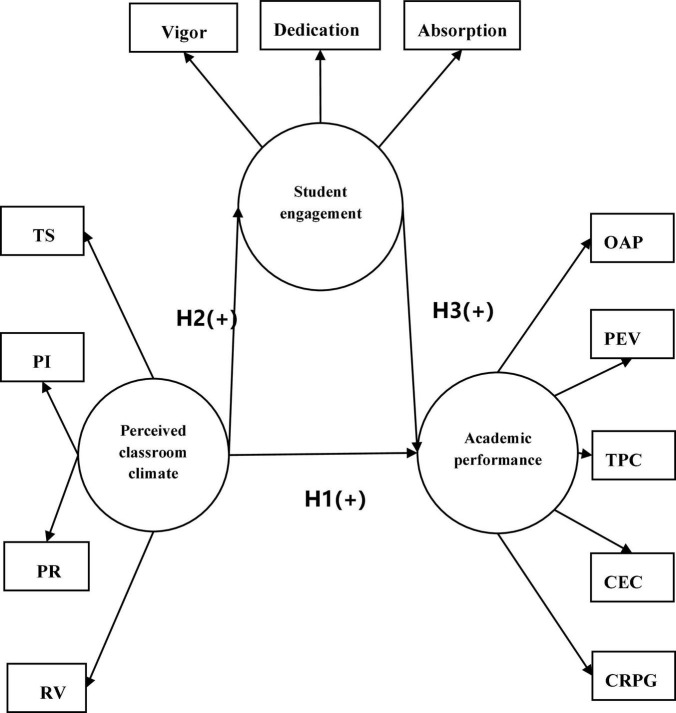
Research hypotheses. TS-academic support from teachers, PI-promoting interaction, PR-promoting mutual respect, RV-respect for viewpoints, OAP-overall academic performance, PEV-professional ethics values, TPC-teaching practice competence, CEC-comprehensive education competence, CRPG-competence in reflection and professional growth.

According to the study demands-resources (SD-R) model, study resources promote student engagement and produce positive study outcomes (Lesener et al., 2020). Study resources include personal resources (such as self-efficacy, psychological resilience, etc.) and environmental resources (such as perceived social support, perceived class atmosphere, etc.). Study outcomes include growth and development in academic performance, mental health, and other areas of growth and development. In the present study, perceived classroom climate can be regarded as one of the environmental resources, student engagement as one of the personal resources, and academic performance as one of the study outcomes. According to Li et al. (2021a), as one of the facets of school environments, school discipline (e.g., good structure, teacher-support, and good teacher-student relationship) is related to better personal resources such as self-control. It follows that perceived classroom climate (environment resource) may promote student engagement (personal resource) and produce positive academic performance (study outcome). Therefore, this study aimed to explore English-major teacher education students’ academic performance (study outcome) in relation to perceived classroom climate (study resource) and further examine student engagement as a mediator between these two variables. The research framework is presented in Figure 1.

### Research hypotheses

#### Perceived classroom climate and academic performance

Researchers have proposed diverse operational definitions of classroom climate. Nevertheless, these definitions all relate to teacher-student interactions (Wang et al., 2020). According to Li et al. (2021b), good teacher-student relationships are associated with high levels of freshmen’s sense of meaning in life, which facilitate their academic adaption. He argued in a recent study that close, supportive, satisfying teacher-student relationships are crucial to college freshmen’s academic adaptation (Li, 2022). According to Vahala and Winston (1994), different perceptions of classroom climate lead to different academic achievements. Namely, perceived classroom climate has a significant association with students’ academic performance (Jafari and Asgari, 2020). For example, a classroom climate focusing on the learning process is beneficial to students’ academic performance, while a classroom climate focusing on learning results is detrimental to students’ academic performance; a supportive, autonomous classroom climate has a positive association with academic performance (Gutiérrez et al., 2019). Other studies have shown that the classrooms constructed with mastery goals (Mirzaei-Alavijeh et al., 2016), more engaging classrooms (Fuqua et al., 2019), and task-centered classrooms that support student autonomy (Lüftenegger et al., 2015) have a significant positive association with academic performance. In addition, in online classrooms and flipped classrooms, independent and cooperative classroom climates are positively related to college students’ academic performance (Estrada et al., 2019; Gao, 2021). Based on the previous studies, we proposed hypothesis 1 of this study:

**H1:** Perceived classroom climate is positively associated with academic performance.

#### Perceived classroom climate and student engagement

According to Guangbao and Timothy (2021), classroom climate is positively associated with student engagement. Students reported in a recent study that a caring, varied, engaging, and well-organized classroom climate with positive and personalized feedback and timely assessment of progress, more actively engages them in their study (Weeldenburg et al., 2021). The perceived classroom climate focusing on the learning process, which pays more attention to interaction, is beneficial to student engagement, while a classroom climate focusing on learning results is detrimental to student engagement (Gutiérrez et al., 2019). The collaborative and inclusive classroom climate makes students more engaged in learning (Sánchez-Hernández et al., 2018). In addition, positive classroom environments, such as engaging students in learning activities (Sakellariou and Tsiara, 2020) and a caring classroom climate (Song and Kim, 2016), help promote student engagement by developing their self-efficacy and sense of belonging (Dogan, 2015). Based on these studies, we proposed hypothesis 2 of this study:

**H2:** Perceived classroom climate is positively associated with student engagement.

#### Student engagement and academic performance

It has been demonstrated that student engagement is positively associated with academic performance. Overall student engagement, emotional engagement, and cognitive engagement are positively associated with academic performance, and emotional engagement has the most explanatory power for academic performance (Sukor et al., 2021). Academic performance, in turn, has a positive association with student engagement. For example, Palos et al. (2019) argued that high academic performance is associated with high student engagement. In addition, the relationship between teaching methods, learning environment, socioeconomic status and academic performance are all mediated by student engagement. For example, the case method of instruction improves students’ academic performance by enhancing their engagement (Song et al., 2022). A study on day or boarding students’ academic performance shows that whether students live in school or not, the critical factor related to their academic performance is student engagement (Simpson and Burnett, 2019). Student engagement has a mediating effect on the relationship between socioeconomic status and academic achievement, so it is an essential factor related to academic performance (Tomaszewski et al., 2020). Based on the previous studies, we proposed hypothesis 3 of this study:

**H3:** Student engagement is positively associated with academic performance.

As mentioned above, perceived classroom climate promotes student engagement, which in turn improves academic performance. Therefore, it is reasonable to assume that student engagement may act as a mediator between perceived classroom climate and academic performance. Though few studies have explored the mediating effect of student engagement on the relationship between perceived classroom climate and academic performance, some studies have indirectly suggested that student engagement may act as a mediator between perceived classroom climate and academic performance. According to Palos (2018), teachers can promote student engagement and ultimately improve students’ academic performance by optimizing the learning process. Promoting perceived classroom climate may be considered one of the optimization tactics. Therefore, we proposed hypothesis 4 of this study:

**H4:** Student engagement mediates the association between perceived classroom climate and academic performance.

## Materials and methods

### Participants

We investigated English-major teacher education students in Hezhou University in Guangxi, China, and in total, 334 questionnaires were collected, and internal consistency checks failed 27 invalid questionnaires, leaving 307 valid samples (91.9%). Among them, 280 (91.2%) were females, and 27 (8.8%) were males, aged 18 to 24 years (*M* = 20.34, SD = 1.26).

### Procedures

A convenience sampling method was adopted to survey English-major teacher education students at Hezhou University in Guangxi, China. The instructor sent the questionnaire link to the students and guided them to complete the questionnaire. An online questionnaire was employed to limit common method variance (CMV) (Tehseen et al., 2017). Informed consent was given by completing and submitting the questionnaire. The cross-sectional survey was conducted anonymously on the platform of WENJUANXING from April 6 to 20.

### Instruments

The measurements in this study contained scales measuring perceived classroom climate, student engagement, and academic performance. This part mainly discusses the composition and measurement of each variable. The questionnaire employed a 5-point Likert scale to measure perceived classroom climate and student engagement, with 1 = *strongly disagree* to 5 = *strongly agree*, and to measure academic performance, with 1 = *very poor* to 5 = *excellent.*

### Perceived classroom climate

This study adopted the Classroom Emotional Climate Scale (Hong et al., 2021) to investigate perceived classroom climate. The perceived classroom climate scale (in English) was translated into Chinese and then translated back to ensure equivalence of meaning. The scale was used to measure students’ perceptions of the supportive, interactive, and respectful climate created by teachers in the classroom. The questionnaire has 12 items with four constructs and three items for each construct. The four constructs are academic support from teachers, promoting interaction, promoting mutual respect, and respect for viewpoints. According to the criteria of Hair et al. (2010), the four constructs fitted the data well (χ2/df = 2.65, RMSEA = 0.07, GFI = 0.93, AGFI = 0.89). The Cronbach’s alpha coefficient of this scale was 0.89 in the study of Hong et al. (2021), and 0.93 in the current study. Items were listed in Appendix.

### Student engagement

This study focused on the overall student engagement, not on the sub-dimensions (vigor, dedication, and absorption), therefore, we chose the ultra-short version of the Utrecht Work Engagement Scale – Student Form (UWES-SF), which was a 3-item version developed by Gusy et al. (2019). Li and Huang (2010) revised the UWES-SF into a Chinese version, and the 3-item version by Gusy et al. (2019) was included in the Chinese version. Therefore, this study adopted the 3-item scale of Gusy et al. translated by Li and Huang (2010). The Cronbach’s α value of this scale was 0.86 in the study of Gusy et al. (2019), 0.61 in the study of Wissing et al. (2022), and 0.88 in this study. Items were listed in Appendix.

### Academic performance

This study referred to the 5-item Academic performance scale of Chinese scholars (Liu et al., 2020) to measure the academic performance of English-major teacher education students, and specific subjects (such as Chinese, mathematics, English, physics, etc.) were replaced with overall academic performance, professional ethics values, teaching practice competence, comprehensive education competence and, competence in reflection and professional growth. The Cronbach’s α value of the scale in this study was 0.89. Items were listed in Appendix.

### Data analysis

AMOS23.0 and SPSS26.0 was used for statistical analyses, and a structural equation model (SEM) was used to evaluate the hypothesis model. After examining the common method variance (CMV), confirmatory factor analysis (CFA) was conducted to test the rationality of the measurement model if the CMV was not serious. The maximum likelihood estimation model parameters and fit indexes were used to test the relationship between the data and the measurement model. As suggested by Hair et al. (2010) for the CFA fitting index, the values of χ2/df, GFI, AGFI, and RMSEA should be calculated. The reliability and validity of the measurement model was tested by the criteria suggested by Hair et al. (2013) for Cronbach’s a reliability coefficient, average variance extracted (AVE), and composite reliability (CR). A bootstrap method was used to examine the mediating effect of student engagement on the relationship between perceived classroom climate and academic performance (Efron, 1992).

### Common method variance

An online questionnaire was employed to limit Common method variance (CMV) (Tehseen et al., 2017). Single factor test suggested by Harman was conducted for CMV of the study variables (Podsakoff et al., 2003). Exploratory factor analysis was conducted for 20 items in the scale, and the results of non-rotated factor analysis were then tested. According to the results, 43% of the explanatory power of the first factor (threshold value: 50%) indicated that the CMV of the variables in this study was not serious.

### Analysis of reliability

According to Hair et al. (2013), Cronbach’s α and composite reliability (CR) values should be higher than 0.70. In this study, Cronbach’s α values of the three scales for perceived classroom climate, student engagement, and academic performance were 0.93, 0.88, and 0.89, and CR values were 0.94, 0.89, and 0.89, respectively, which showed that the measurement tool had good reliability.

### Analysis of validity

Based on the suggestions of Fornell and Larcker (1981), the criterion for evaluating convergent validity is that the higher the factor loading value is, the higher the convergent validity is. The factor loading value should be at least 0.50. In the current study, factor loadings ranged from 0.62 to 0.92. Factor loadings of items can be seen in Appendix.

As suggested by Hair et al. (2013), the acceptable value of average variation extraction (AVE) should be greater than 0.50. In this study, the AVE values of perceived classroom climate, student engagement, and academic performance scales were 0.57, 0.74, and 0.63, respectively, as shown in Table 1. According to Fornell and Larcker (1981), the square root of the average variation extraction (AVE) of a construct should be greater than its correlation coefficients, which indicates that the construct has good discriminant validity. In this study, the square root of each construct’s AVE was greater than all its correlation coefficients, as shown in Table 2.

**TABLE 1:** Analysis of validity and reliability.

Contact	Cronbach α	CR	AVE	FL
Threshold	>0.70	>0.70	>0.50	>0.50
Perceived classroom climate	0.93	0.94	0.57	0.67∼0.88
Student engagement	0.88	0.89	0.74	0.73∼0.92
Academic performance	0.89	0.89	0.63	0.62∼0.89

CR-composite reliability; AVE-average variation extraction; FL-factor loading.

**TABLE 2:** Correlation coefficients and discriminant validity analysis.

Construct	M ± SD	Maximum	1	2	3
1 Perceived classroom climate	4.11 ± 0.66	5	**0.75**		
2 Student engagement	3.61 ± 0.77	5	0.50*	**0.86**	
3 Academic performance	3.52 ± 0.63	5	0.26*	0.41*	**0.79**

*Represents p < 0.001; the figures in bold are the square root of the average variation extraction (SQAVE).

## Results

### Model fit analysis

According to Schreiber et al. (2006), in an acceptable model, χ^2^/df should be less than 3, RMSEA should be less than 0.08, NFI, NNFI, and GFI higher than 0.95, and CFI, IFI higher than 0.95. The findings in this study were as follows: χ^2^/ df = 2.06,RMSEA = 0.06, NFI = 0.96, NNFI = 0.97, GFI = 0.95, CFI = 0.98, IFI = 0.98, which indicated that the model fitting was acceptable.

### Direct effects analysis

**FIGURE 2:**
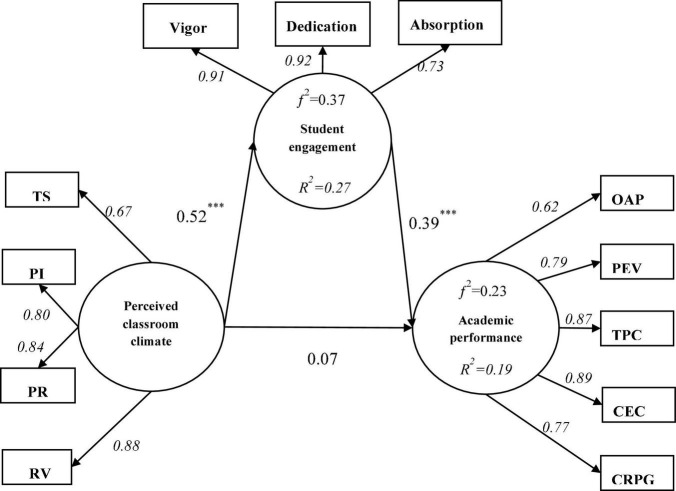
Verification of the research model.

Bootstrapping technique was adopted to test the direct effect among the variables and 5,000 samples were repeated for testing (Efron, 1992). The bias-corrected percentile bootstrap method was used to test the 95% confidence interval. The confidence interval does not contain zero, indicating that there is a direct effect between variables, otherwise, indicating that the direct effect is not significant. Direct effects can be seen in Figure 2 and Table 3.

**TABLE 3:** Direct and indirect effects analysis.

Parameter	Estimate	Lower bounds	Lower bounds
**Standard direct effects**			
PCC→AP	0.07	−0.06	0.21
PCC→SE	0.52*	0.42	0.62
SE→AP	0.39*	0.25	0.53
**Standard indirect effects**			
PCC→SE→AP	0.21*	0.13	0.31

*The empirical 95% confidence interval does not contain zero.

PCC, perceived classroom climate; SE, student engagement; AP, academic performance.

Perceived classroom climate was not significantly associated with academic performance (β = 0.07, *P* > 0.05) because confidence intervals (−0.06, 0.21) contained zero. After controlling the effect of student engagement, perceived classroom climate had a significant association with academic performance (β = 0.28, *p* < 0.001), as the confidence interval (0.16, 0.39) did not contain zero. Thus, hypothesis 1 was supported. That is, perceived classroom climate was positively associated with academic performance.

Perceived classroom climate had a positively significant association with student engagement (β = 0.52, *p* < 0.001), as the confidence interval (0.41, 0.62) did not contain zero. Thus, hypothesis 2 was supported. That is, perceived classroom climate was positively associated with student engagement.

The confidence interval (0.25, 0.53) did not contain zero, indicating student engagement had a positively significant association with academic performance (β = 0.39, *p* < 0.001). Thus, hypothesis 3 was supported. That is, student engagement was positively associated with academic performance.

Hair et al. (2011) stated that the value of *R*^2^ is explained as the exogenous latent variables’ combined effects on the endogenous latent variable. *R*^2^-values of 0.75, 0.50, or 0.25 indicate significant, moderate, or weak determination coefficients, respectively. The explanatory power of perceived classroom climate for student engagement is 27%; the explanatory power of perceived classroom climate and student engagement for academic performance is 19%, as shown in Figure 2.

Furthermore, the *f*^2^ effect size value represents the contribution of the exogenous variable to *R*^2^ values of the endogenous variable (*f*^2^ = *R*^2^/(1− *R*^2^)) (Cohen, 1992). The *f*^2^ effect size values of 0.02, 0.15, and 0.35 indicate small, moderate, and significant effects of the exogenous latent variable, respectively. Student engagement was explained by perceived classroom climate with an effect size *f*^2^ of 0.37, thus indicating a significant effect size. Academic performance was explained by perceived classroom climate and student engagement with an effect size *f*^2^ of 0.23, thus indicating a medium effect size, as shown in Figure 2.

### Mediating effect analysis

Adopting the bootstrap method, this study examined the mediating effect of student engagement with 5000 repeated samples (see Figure 2). The indirect effect of student engagement as the mediating variable was 0.21 (95% CI = [0.13, 0.31], *p* = 0.000), indicating that the mediating effect of student engagement was significant. The direct effect of perceived classroom climate on academic performance was 0.07 (95% CI = [−0.05, 0.21], *p* = 0.265), indicating no direct effect of perceived classroom climate on academic performance. As Preacher and Hayes (2004) suggested, complete or partial mediation rely on the simple mediation model (X→M→Y), constraining the X→Y path to zero. If the χ^2^ statistic is significant, then constraining the X→Y path to zero is regarded as unreasonable given the data, ruling out the possibility of complete mediation by Baron and Kenny (1986). After constraining the perceived classroom climate (X)→academic performance (Y) path to zero, the χ^2^ statistic is significant (χ^2^ = 106.06, *p* = 0.000). Specifically, student engagement acted as a partial mediator in the relationship between perceived classroom climate and academic performance. Thus, hypothesis 4 was supported.

## Discussion

This study objective was to explore the characteristics of teacher education students’ academic performance and its associations with perceived classroom climate and student engagement, and examine the effect of student engagement on the relationship between the other two variables.

### Perceived classroom climate and academic performance

We found that perceived classroom climate was positively associated with academic performance. Perceived classroom climate is an essential factor related to students’ academic performance in various student groups (Vahala and Winston, 1994). Perceived classroom climate has been found to have a direct and significant relation with academic performance among undergraduates and postgraduates (Jafari and Asgari, 2020). Independent and cooperative classroom climate in an online and flipped classroom has a positive association with the academic performance of college students (Estrada et al., 2019; Gao, 2021). The academic performance of mathematics majors is positively associated with the supportive and task-centered classroom climate (Lüftenegger et al., 2015). A study among medical students reported that the class climate with mastery goal orientation is positively associated with their academic performance (Mirzaei-Alavijeh et al., 2016). It was also found among middle school students that class climate oriented by mastery goals is positively associated with students’ academic performance (Gutiérrez et al., 2019). Fuqua et al. (2019) argued that a more engaging classroom climate for engineering students leads to better academic results. Consistent with these findings, this study also found that perceived classroom climate, such as academic support from teachers, promoting interaction and mutual respect, etc., improved academic performance of English-major teacher education students.

In terms of the status of perceived classroom climate, Hong et al. (2021) took students from three universities in northern Taiwan as research samples and found that their scores on perceived classroom climate were above the average. Wang et al. (2020) also found an above-average score on classroom climate among medical students in China. Consistent with the prior studies, the score of perceived classroom climate among English-major teacher education students was also above average. However, among nursing students of a university in Trabzon, Kurt et al. (2022) found that scores on classroom climate were below the average. This discrepancy may result from survey samples of different majors. Moreover, the score on English-major teacher education students’ academic performance was also above average. As the teacher education students’ academic performance scale used in this study was newly developed, there is a lack of relevant data in previous studies. Thus, we look forward to more studies in the future using this tool to measure the academic performance of teacher education students.

### Perceived classroom climate and student engagement

This study revealed that perceived classroom climate was positively associated with student engagement. Students prefer a caring, varied, challenging, fulfilling, engaging, well-organized classroom climate with positive and personalized feedback and timely assessment of progress (Weeldenburg et al., 2021). For example, teachers’ cooperative and inclusive classroom climate makes students more engaged in learning (Sánchez-Hernández et al., 2018). In addition, positive classroom environments, such as engaging students in learning activities (Sakellariou and Tsiara, 2020) and a caring classroom climate (Song and Kim, 2016), help promote student engagement by developing their self-efficacy and sense of belonging (Dogan, 2015). Consistent with these findings, English-major teacher education students also preferred a supportive (academic support from teachers), interactive and cooperative (promoting interaction), and respectful and inclusive (promoting mutual respect and respecting viewpoints) classroom climate in which they showed higher levels of student engagement.

As for the status of student engagement, most of the studies have reported a moderate level of student engagement among university or college students. For example, Dimitriadou et al. (2021) found a moderate level of student engagement among university students in Greek. A survey of undergraduate medical students in Dutch indicated a moderate level of student engagement (Wissing et al., 2022). A moderate level of student engagement was also found among university students in Germany (Körner et al., 2021; Teuber et al., 2021). Not exactly the same as the above research results, the score on student engagement of our sample was above average. This discrepancy may result from the fact that our samples are teacher education students, who, as future teachers, are expected to be more engaged in their studies.

### Student engagement as a mediator

The result of this study showed that student engagement was positively associated with academic performance, and the effect of perceived classroom climate on academic performance was partially mediated by student engagement. Studies have demonstrated that student engagement significantly predicts college students’ academic performance (Sukor et al., 2021). In addition, Tomaszewski et al. (2020) argued that student engagement mediates the relationship between students’ socioeconomic status (SES) and academic achievement. Other studies have demonstrated that teaching methods and learning environment are factors related to academic performance, and the relationships between these variables and academic performance are mediated by student engagement (Simpson and Burnett, 2019; Song et al., 2022). This study found that classroom climate perceived by English-major teacher education students improved their academic performance by promoting their student engagement, suggesting that student engagement was an important factor associated with academic performance, which supported the findings of previous studies.

### Contributions of this study

As discussed above, the findings of this study supported all the hypotheses. According to the motivation process of the study demands-resources (SD-R) model, study resources promote student engagement and produce positive study outcomes (Lesener et al., 2020). In the present study, perceived classroom climate was regarded as one of the environmental resources, student engagement as one of the personal resources, and academic performance as one of the study outcomes. The findings of this study revealed that perceived classroom climate (an environmental resource) promoted student engagement (a personal resource) and academic performance (a study outcome), and student engagement (a personal resource) improved academic performance (a study outcome). Specifically, student engagement partially mediated the relationship between perceived classroom climate and academic performance. These findings supported the motivation process of the SD-R model, which is in line with the results of Tomaszewski et al. (2020).

This study was conducted among the English-major teacher education students to explore their academic performance in relation to perceived classroom climate and student engagement. Although prior studies have adopted samples of different student groups, including secondary school students, to explore the mediating role of student engagement between various variables, few studies have examined its mediating role between perceived classroom climate and academic performance, especially among English-major teacher education students. Therefore, this study fills the gap in the research on the associations between these three variables, with a sample of English-major teacher education students.

### Practical suggestions

As revealed in this study, perceived classroom climate and student engagement are significant factors related to students’ academic performance. Therefore, teachers should create a positive classroom climate and promote student engagement of teacher education students, so as to improve their academic performance. In teacher education reform, teachers and educators are encouraged to adopt effective strategies and technologies to create a supportive, interactive, cooperative, respectful, and inclusive classroom climate, in which students have positive classroom experiences and thus a high level of student engagement. Through their own learning experiences, English-major teacher education students become aware of the significance of classroom climate and student engagement for all students, including secondary school students. As teacher education students are future teachers, it is an important aspect of their academic performance to know how to create a positive classroom climate and promote student engagement. In the training of their teaching skills, teacher education students are encouraged to develop their competencies in teaching practice through professional learning or by imitating the way their teachers create an inclusive and respectful climate and express support and care in class (Tsai and Ku, 2021). In addition, teachers and educators should guide teacher education students to understand the factors linked to student engagement and grasp strategies to promote student engagement of secondary school students.

### Limitations and future study

The samples selected in this study were only English-major teacher education students at Hezhou University in Guangxi, China, and therefore the research results are not representative enough. Future studies should adopt samples of teacher education students from different majors, colleges, and regions to enhance the generalization of results.

In addition, this cross-sectional study could not establish a causal relationship between perceived classroom climate, student engagement, and academic performance. Future research should carry out longitudinal studies to explore the causal relationship between these variables.

Moreover, although perceived classroom climate had a positive association with student engagement, there may be other factors related to student engagement in terms of classroom teaching, such as students’ personality traits (ego, values, etc.). Therefore, future research should examine other factors in relation to perceived classroom climate and student engagement.

## Conclusion

The findings of this study supported the hypotheses. That is, the perceived classroom climate was positively associated with student engagement and academic performance among English-major teacher education students in Guangxi, China; their student engagement was positively associated with their academic performance, and student engagement partially mediated the association between perceived classroom climate and academic performance.

From the above findings, it is reasonable to conclude that perceived classroom climate and student engagement are critical factors related to the academic performance of English-major teacher education students. The former improves their academic performance through the mediating effect of the latter. The findings may help explore practical ways for teacher education reform; or rather, teacher educators are advised to find ways to promote students’ academic performance by improving classroom climate and increasing student engagement.

## Data availability statement

The original contributions presented in this study are included in the article/supplementary material, further inquiries can be directed to the corresponding author.

## Ethics statement

Ethical review and approval were not required for the study on human participants following the local legislation and institutional requirements. Informed consent of the participants was given by completing and submitting the questionnaire.

## Author contributions

YM and CW: concept and design, drafting of the manuscript, acquisition of data, statistical analysis, and critical revision of the manuscript. Both authors contributed to the article and approved the submitted version.

## Conflict of interest

The authors declare that the research was conducted in the absence of any commercial or financial relationships that could be construed as a potential conflict of interest.

## Publisher’s note

All claims expressed in this article are solely those of the authors and do not necessarily represent those of their affiliated organizations, or those of the publisher, the editors and the reviewers. Any product that may be evaluated in this article, or claim that may be made by its manufacturer, is not guaranteed or endorsed by the publisher.

## Appendix

**TABLE A1:** Items and factor loadings of the perceived classroom climate scale, student engagement scale and academic performance scale.

Item	Factor loading
**Perceived classroom climate scale (12 items)**	
**Factor 1→academic support from teachers**	
(1). The teacher is concerned about how much I have actually learned (老师关心我到底学到了多少	0.84
(2). The teacher wants me to do my best at my school work (*老师想让我在学习上做到最好*)	0.84
(3). The teacher wants to help me learn (*老师想要在学习上帮助我*)	0.87
**Factor 2→promoting interaction**	
(4). The teacher encourages us to share our views with other people in the classroom (*老师鼓励我们在课堂上分享观点*)	0.92
(5). The teacher encourages the classmates to understand each other (*老师鼓励同学们相互理解*)	0.83
(6). The teacher encourages us to help with the work of other classmates (*老师鼓励我们帮助其他同学学习*)	0.81
**Factor 3→promoting mutual respect**	
(7). The teacher does not allow students to laugh at the ideas of others (*老师不允许同学们嘲笑他人想法*)	0.84
(8). If someone answers a question incorrectly in class, the teacher will not let classmates laugh at him or her (*老师不允许同学们嘲笑答错问题的人*)	0.81
(9). The teacher does not permit classmates to say bad things about each other (*老师不允许同学们说彼此坏话*)	0.92
**Factor 4→respecting viewpoints**	
(10). The teacher will respect student views (*老师会尊重学生的观点*)	0.85
(11). In class, classmates will not obstruct the outstanding performance of others (*在课堂上, 同学们不会妨碍他人的优秀表现*)	0.81
(12). In class, classmates do not have to worry about the pressure of having to express opinions (*在课堂上, 同学们不必担心必须发表意见*)	0.70
**Student engagement scale (3 items)**	
(13). When I’m doing my work as a student, I feel bursting with energy (*学习时, 我感到精力充沛*)	0.91
(14). I am enthusiastic about my studies (*我对学习充满热情*)	0.92
(15). I am immersed in my studies (*学习时, 我忘了周围的一切*)	0.73
**Academic performance scale (5 items)**	
(16). Compared with my classmates, my overall academic achievement is (*与我的同学相比, 我的总体学业成就*)	0.62
(17). Compared with my classmates, my identification and practice of professional ethics values are (*与我的同学相比, 我的师德践行能力*)	0.79
(18). Compared with my classmates, my teaching practice competence is (*与我的同学相比, 我的教学实践能力*)	0.87
(19). Compared with my classmates, my comprehensive education competence is (*与我的同学相比, 我的综合育人能力*)	0.89
(20). Compared with my classmates, my competence in reflection and professional growth is (*与我的同学相比, 我的反思发展能力*)	0.77
